# 1136. Effect of Weekly Antibiotic Rounds as a Core Strategy of the Antimicrobial Stewardship Program on Antibiotic Utilization in a Terciary-care Neonatal Intensive Care Unit, Medellin, Colombia

**DOI:** 10.1093/ofid/ofab466.1329

**Published:** 2021-12-04

**Authors:** Alejandro Diaz Diaz, Juan Gonzalo Mesa-Monsalve, Adriana M Echavarria-Gil, Carolina Jimenez

**Affiliations:** 1 Hospital General de Medellin, Medellin, Antioquia, Colombia; 2 Hospital General de Medellin/Clínica Las Américas Auna, Envigado, Antioquia, Colombia

## Abstract

**Background:**

Antibiotics are among the most prescribed drugs in the neonatal intensive care unit (NICU), but frequently are used inappropriately exposing preterm neonates to additional harm. Antibiotic stewardship programs (ASP) have demonstrated impact on antibiotic use in the hospital setting, but implementation in neonatal units is challenging. We sought to determine the effects of weekly antibiotic rounds on overall antibiotic consumption in the NICU.

**Methods:**

Single-center, retrospective observational study. In November 2014, we implemented weekly antibiotic rounds in a 60-bed tertiary-care NICU, led by a pediatric infectious disease physician. Antibiotic therapy decisions were made in collaboration with neonatologists. Data collected included the proportion of patients receiving antibiotics, irrespective of the indication. Multimodal ASP was implemented hospital-wide in 2015. Antibiotic consumption was measured with days of therapy (DOT). Data on costs and in-hospital mortality were obtained from pharmacy and hospital records.

**Results:**

From November 2014 to December 2020, we evaluated 13609 neonates admitted to the NICU during rounds. Of those, 3607 (27%) were receiving at least one antibiotic. Overall, the proportion of patients with antibiotics decreased from 31% to 19% during the study period (p< 0.001). In 2017, an outbreak of neonatal necrotizing enterocolitis (NEC) occurred. Specific countermeasures as well as reinforcement of ASP were implemented. Despite Antibiotic usage by DOT increased in 2017 driven by empiric treatment with piperacillin tazobactam in patients with NEC, overall antibiotic consumption decreased from 254.4 DOT/1000 patient days (PD) to 162.4 DOT/1000 PD (Figure 1). Annual costs from antibiotic prescriptions were US&23,161 in 2015 and decreased to US&12.046 in 2020 saving over US&3,800/year (fig 2a). During the study period, we did not observe an increase in crude in-hospital mortality rate (Figure 2b).

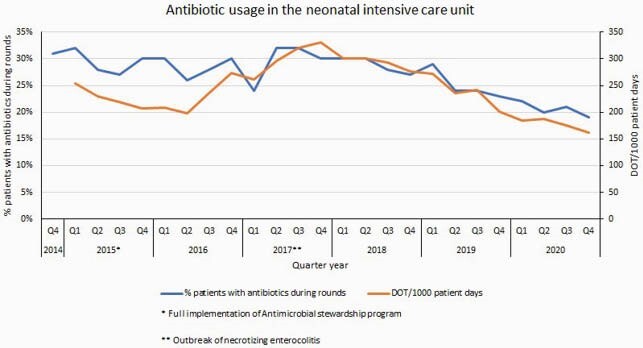

Primary Y axis indicates the proportion of patients with at least one antibiotic prescription during rounds. Secondary Y axis indicates antibiotic consumption by days of therapy metrics.

Antibiotic prescription costs and NICU mortality rates during study period

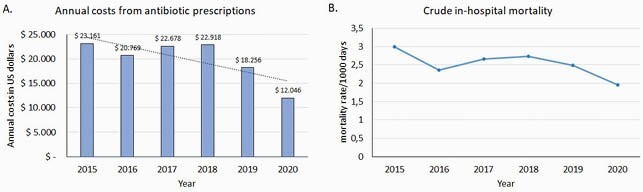

A. Annual antibiotic prescription costs; B. NICU mortality rate

**Conclusion:**

Weekly antibiotic rounds led to a significant decrease in antibiotic utilization in our NICU. This strategy is relatively simple and low-cost, saves hospital resources and has a large impact on antibiotic use. Hence, its implementation is encouraged as part of successful antimicrobial stewardship programs.

**Disclosures:**

**All Authors**: No reported disclosures

